# Drifting Phenologies Cause Reduced Seasonality of Butterflies in Response to Increasing Temperatures

**DOI:** 10.3390/insects9040174

**Published:** 2018-11-30

**Authors:** Zachariah J. Gezon, Rebekah J. Lindborg, Anne Savage, Jaret C. Daniels

**Affiliations:** 1 Conservation Department, Disney’s Animals, Science, and Environment, Lake Buena Vista, FL 32830, USA; zgezon@thanksgivingpoint.org (Z.J.G.); asavage@proyectotiti.com (A.S.); 2 Thanksgiving Point Institute, Lehi, UT 84043, USA; 3 Florida Museum of Natural History, Gainesville, FL 32611-2710, USA; jdaniels@flmnh.ufl.edu; 4 Entomology and Nematology Department, University of Florida, Gainesville, FL 32611-2710, USA

**Keywords:** climate change, citizen science, butterfly, community composition, phenology

## Abstract

Climate change has caused many ecological changes around the world. Altered phenology is among the most commonly observed effects of climate change, and the list of species interactions affected by altered phenology is growing. Although many studies on altered phenology focus on single species or on pairwise species interactions, most ecological communities are comprised of numerous, ecologically similar species within trophic groups. Using a 12-year butterfly monitoring citizen science data set, we aimed to assess the degree to which butterfly communities may be changing over time. Specifically, we wanted to assess the degree to which phenological sensitivities to temperature could affect temporal overlap among species within communities, independent of changes in abundance, species richness, and evenness. We found that warming winter temperatures may be associated with some butterfly species making use of the coldest months of the year to fly as adults, thus changing temporal co-occurrence with other butterfly species. Our results suggest that changing temperatures could cause immediate restructuring of communities without requiring changes in overall abundance or diversity. Such changes could have fitness consequences for individuals within trophic levels by altering competition for resources, as well as indirect effects mediated by species interactions across trophic levels.

## 1. Introduction

Climate change is affecting species globally through range shifts [1,2], species invasions [3,4], extinctions [5,6], and altered phenology [3,7]. Altered phenology is among the most commonly described ecological consequences to climate change, and altering phenology can have effects on survival and reproduction. Phenological events are typically strongly influenced by certain abiotic factors, such as temperature [8,9], precipitation [8,10], and photoperiod [8,11]. Moreover, more than one of these factors may influence phenologies simultaneously. For example, the flowering phenology of wildflowers in the Rocky Mountains is best explained by a combination of the date of bare ground and soil temperature [12]. Each species within a community can have unique requirements to the abiotic cues influencing phenology [13,14]. Thus, changes in abiotic cues could influence species within a community differently, causing changes in the temporal overlap of species and affecting species interactions [15,16]. Species interactions among insects with short flight seasons may be particularly sensitive to changes in phenological mismatches. For example, small sweat bees (*Halictidae*) may only fly for two to three weeks per season, and a phenological mismatch between the bee and a floral resource of just one day could cause an almost 10% reduction in the bee’s opportunity to forage before nest provisioning begins [16].

Studies on the ecological consequences of climate change often focus on single species, or tend to focus on pairwise species interactions at most [17,18,19]. However, most ecological systems are comprised of a diverse suite of organisms, with ecologically similar species within trophic levels (e.g., the dozens of species of wild flowers or butterflies in a meadow). Recent studies are exploring how multiple species are affected differently by the same shifts in abiotic factors [14,15,20,21,22]. However, the emphasis of these studies tends to focus on there being differences or similarities among species within a community, and tend not to focus on the specific community changes that happen as a consequence of climate change. Understanding how suites of species respond to climate change in concert and considering the potential ecological consequences of such changes is therefore a logical next step in understanding how communities may respond to climate change over the coming decades.

Subtropical systems differ from temperate systems in that seasonality is less punctuated by dramatic abiotic changes, and diverse communities are often present during all months of the year [23]. In many temperate systems, snowmelt signals the beginning of the growing season of most plants, and soon after, the flight season of many insects. Within the flight season, there is a typical succession of species. For example, spring beauties and glacier lilies are typically the first flowers to bloom after the snow melts in the Rockies [12,24], and Aspen sunflowers are typically the last flowers standing in the fall [24]. In such communities, there is very little opportunity for the early-season species to overlap in time with the late-season species. In contrast, seasons in tropical and subtropical habitats may be less clearly constrained, and some species can be present year-round or have more than one flight per year [23,25]. Thus, subtropical systems offer the opportunity for more phenological drift in either direction (earlier or later in the year), to the point where “early” and “late” season species could find themselves being active simultaneously. Conventional wisdom tends to posit that seasonal timing is more important in temperate regions. However, tropical and subtropical habitats may be more open to fluid phenological windows than temperate regions due to the increased effects of thermal change [26,27], and thus, tropical and subtropical regions may be more sensitive to phenological asynchronies.

To investigate how species assemblages in a subtropical climate may be responding to a changing climate, we analyzed a twelve-year (2005–2016) data set collected from four surveyed butterfly routes within Walt Disney World^®^ property, in Orange County, FL, USA. We predicted that the phenologies of each species of butterfly would have unique responses to the abiotic environment, which would compound to cause changes in butterfly community composition over time, even in the absence of changes in butterfly species’ richness, evenness, or the population size of individual species. Specifically, we hypothesized that the flights of the various species would shift relative to one another, creating unique communities flying at any given time.

## 2. Materials and Methods

### 2.1. Study System

This study was conducted in the Wildlife Management and Conservation Area (WMCA) on Walt Disney World Resort^®^ property in Lake Buena Vista, Florida (Orange County) from 2004–2016. The WMCA consists of approximately 3370 ha of privately accessed wetland and upland habitat, and is designated as a permanent conservation area. Habitat types in the WMCA consisted of oak scrub, pine flatwoods, and temperate hardwood forest. The study site is subtropical, with temperatures typically ranging from −3 °C to 37 °C throughout the year (National Oceanic and Atmospheric Admiration [NOAA] weather station KISSIMMEE 2 FL US #84625). We also obtained long-term temperature and precipitation data collected at Orlando International Airport (MCO) from the Florida State University’s Florida Climate Center (NOAA weather station #12815) to look at broad climate patterns in our area over the past five decades. The hurricane season occurs from June through November, with some areas in the WMCA flooding during these months and remaining flooded for long periods of time. Monthly average rainfall ranges from 19.18 cm at its peak in June during hurricane season to 3.76 cm during the driest month of December (NOAA weather station KISSIMMEE 2 FL US #84625).

### 2.2. Sampling Design

**Figure 1:**
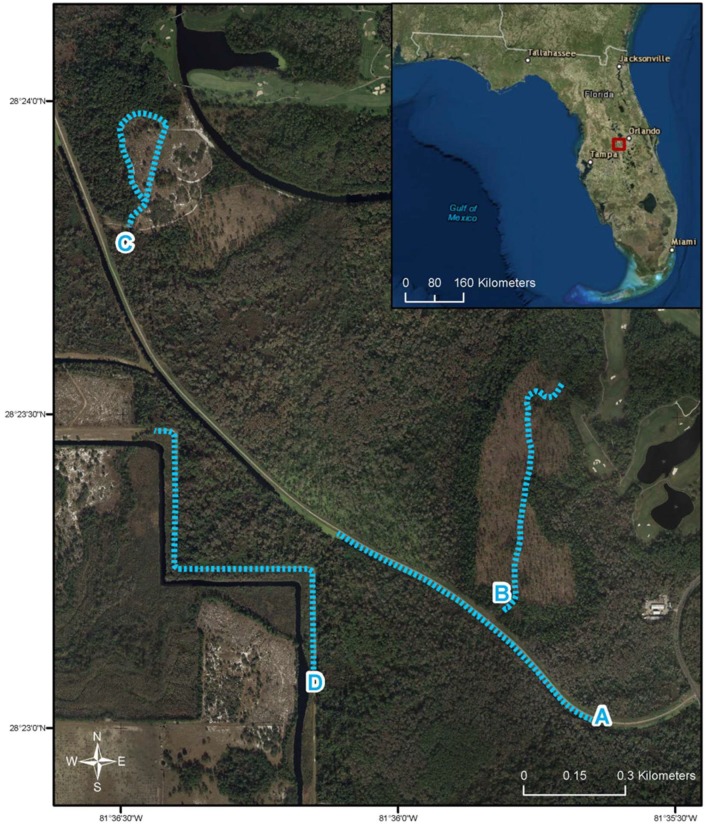
Site map of the study area within Walt Disney World Resort^®^’s Wildlife Management and Conservation Area, showing the four sampling routes used throughout this study.

We used a total of four designated butterfly monitoring sites (Routes A–D, Figure 1) in this study. Routes were chosen based on proximity to access roads and to be representative of local habitat types. Routes A and D were roadside habitats bordering patches of pine forest, scrub habitat, and wetland habitat. Routes B and C were near roadways but ran within pine forest, scrub, and wetland habitats. Routes ranged in length from 0.64 km to 1.12 km, and the average distance between routes was 0.64 km (Arc GIS 10, Esri, Redlands, CA, USA).

We sampled each route monthly between 1000–1200 or 1400–1600 hr, and on days with less than 50% cloud cover and light to moderate wind using the non-invasive method developed by Pollard (1975, 1977). Routes were walked by an observer at a slow and uniform pace, observing and recording all butterflies sighted ahead and to the sides of the observer to a maximum distance of 5 m. Each survey of a single route on a single day (hereafter referred to as a “bout”) was surveyed by teams consisting of a trained observer, plus zero to two additional participants to assist with data collection and insect identification. Butterflies were assumed to be absent from overgrown areas that were unable to be seen, such as tall willows running along a route [28,29]. Estimates of wind speed and percent cloud cover were recorded at the beginning of each sampling bout. Sample bouts were canceled or rescheduled in the case of rain or cloud cover greater than 50%. The start and stop times of each sample bout were recorded and used to determine sampling effort (in minutes). Sampling was paused to chase, capture, and identify fast-moving or elusive individuals as needed. Unidentifiable butterflies were recorded as “unidentified” along with the lowest taxonomic resolution possible (e.g., “unidentified skipper”).

### 2.3. Analyses

Weather data: Using long-term data collected at Orlando International Airport, we calculated the thermal sum for each year by totaling the mean temperature for each day in each calendar year. We also approximated the incidence of frost by determining the number of days per year where the temperature dropped below freezing 0 °C. Although frost is more complex than simply falling below zero [24], freezing temperatures can be an adequate proxy. To determine if either the maximum thermal sum or total number of frost days increased over time, we used simple linear regression with the appropriate response variable as a function of year.

Community composition: To determine how butterfly community composition changed over a twelve-year time span, we calculated the total abundance, species richness, and evenness (calculated as Evar, [30]). Evar ranges from 0 to 1, with 0 representing minimum evenness and 1 representing maximum evenness [30]. We then tested whether these metrics of community composition varied as a function of year using simple linear regression, as log+1 transformed abundance to fulfill the assumptions of the model. We then used non-metric multidimensional scaling (NMDS), using the Bray-Curtis dissimilarity index, to visualize the butterfly communities. NMDS was performed using the vegan package [31] in R version 3.0.3 [32]. Stress values from models with two to eight dimensions were compared to determine the most appropriate summary of the butterfly community.

To determine how community composition varied over the course of each sampling season, we used general linear mixed models. We used corrected Akaike’s Information Criterion (AICc) values to rank and compare multiple *a priori* hypotheses and pick the model best supported by the data. The candidate models included the day of the year, lag precipitation (total precipitation in the 60 days prior to the sampling bout), fixed effects and sampling effort (in minutes), sample route (A–D), wind (ranked 1–5, see Section 2.2), cloud cover (as a percent), and temperature at the start of the sample bout (°C) as random effects. We used the lmer function in the lme4 package to construct the models, and the aictab function in the AICcmodavg package to rank and compare models using R version 3.0.3 [32,33,34]. We used type III sums of squares and the Satterthwaite approximation of degrees of freedom to calculate *F* and *P* values using the lmerTest package [35], and estimated the *R*^2^ values using the sem.model.fits function in the piecewiseSEM package [36,37]. Continuous explanatory variables were rescaled prior to analysis using the rescale function in the scales package [38]. Separate analyses were then performed for each year.

We expected that relatively warm temperatures would spread butterfly activity over a longer period of time, meaning that warm years would be less seasonal than colder years. To test this hypothesis, we used the quadratic term for the day of the year from the aforementioned linear mixed models, and tested whether it varied as a function of annual thermal sum. Likewise, we expected that the seasonal community composition would have a lower predictability (*R*^2^ values) in the warmer years. To test this hypothesis, we used the *R*^2^ values from the aforementioned linear models and tested whether it varied as a function of the annual thermal sum.

## 3. Results

Across 12 years and 4 transects, we conducted 510 sampling bouts with 83 citizen scientists (28 as primary observers, and 55 as participants). Individual citizen scientists conducted anywhere from 1 to 136 sampling bouts (mean = 8.6 bouts per participant). Throughout the study period, 74 species of butterflies were identified, which represents approximately 37% of butterfly species in Florida, and approximately 10% of North American butterflies north of Mexico. Collectively, these insects belonged to 49 genera from 6 families (Table S1). The most commonly sighted butterflies were the white peacock (*Anartia jatrophae)*, fiery skipper (*Hylephila phyleus)*, and palamedes swallowtail (*Papilio palamedes)* (27.7%, 7.6%, and 7.0% of sightings, respectively). We observed 6 species represented by a single individual. 1.1% of the observations were recorded as “unidentified”. We observed 50% of the documented species after just 27 of the sample bouts, and 100% of the species after 299 bouts (i.e., no new species were observed in the final 211 sample bouts). Species accumulation and rarefaction curves of the sampling data (Figures S1 and S2, respectively) revealed a saturating function, suggesting that our efforts adequately characterized the butterfly fauna of the WMCA, and that continued sampling will record new, rarer species at a low rate, if at all.

**Figure 2:**
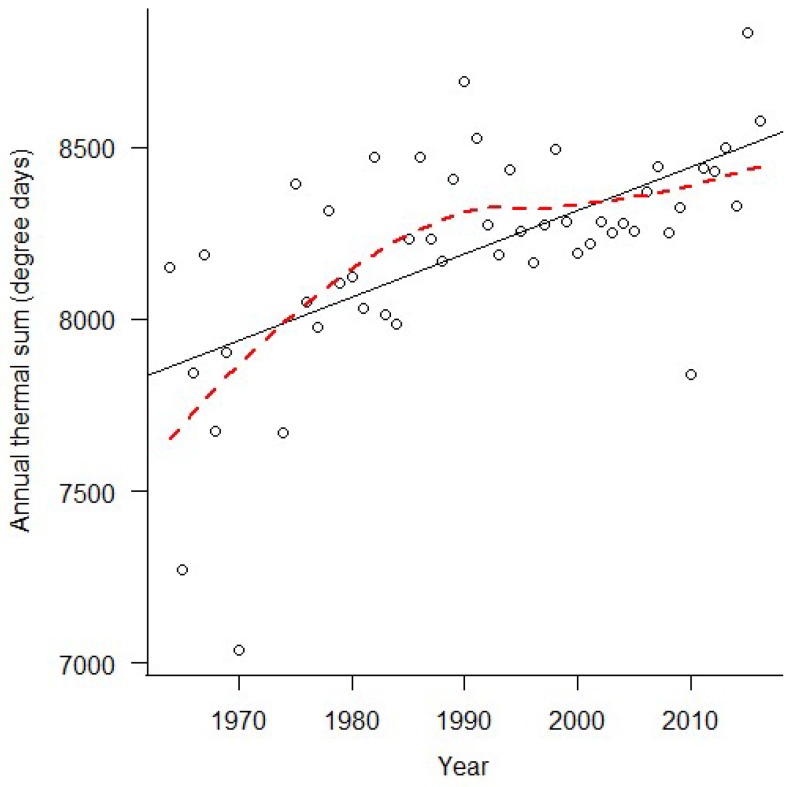
Long-term thermal sum trend. The total amount of heat experienced by organisms in Central Florida is increasing over time. The thermal sum (in degree days, °Cd) was calculated by totaling the mean temperature for each day within each year, and a best-fit line was added (solid black) using simple linear regression. The red dotted line was added using locally estimated scatterplot smoothing (LOESS) with a span of 1 for visualization purposes.

**Figure 3:**
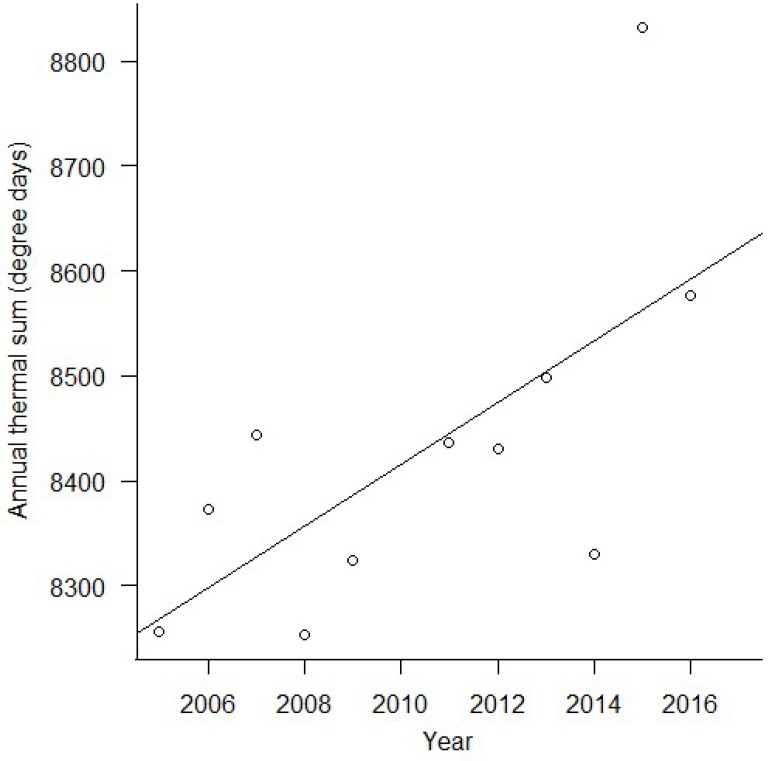
Thermal sum over the sampling period. Thermal sum increased over the course of our sample period (*R*^2^ = 0.39, *F*_1, 9_ = 7.42, *p* = 0.023), shown here without 2010, which was an extremely influential outlier. The relationship is qualitatively similar, although not significant when 2010 is included (Figure S3).

**Figure 4:**
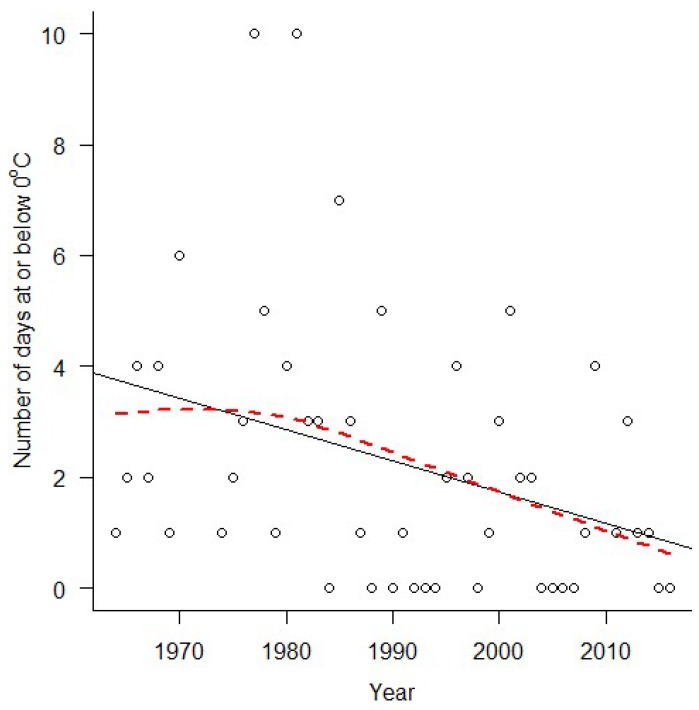
Frost events over time. The number of days per year where the temperature drops below freezing (0 °C) is decreasing over time in Central Florida. Data was obtained from the Florida Climate Center’s website, collected at the Orlando International Airport from 1964 through 2016. This figure excludes 2010, an extreme outlier with 13 days below freezing, due the “polar vortex”. The pattern is similar, though not significant when 2010 is included in the analysis. The red dotted line was added using LOESS with a span of 2 for visualization purposes due to violations of the assumptions of simple linear regression.

Weather data obtained from the Florida State University Climate Center (“Downloadable Data”, 2017) revealed that the annual thermal sum in central Florida has been increasing since data collection began in 1964 (Figure 2, *R*^2^ = 0.35, *F*_1, 48_ = 27.46, *p* < 0.0001). Likewise, the annual thermal sum increased over the 12 sampling years used in the present study (Figure 3, *R*^2^ = 0.39, *F*_1, 9_ = 7.42, *p* = 0.02). In analyses presented in the main manuscript, 2010, the infamous “polar vortex” year, was removed from the dataset as an extreme and influential outlier. When 2010 is included, the results are quantitatively, but not qualitatively, different (i.e., parameter estimates change but our interpretation of the data does not), but meeting the assumptions of the models was more challenging. Analyses with 2010 included are available in the supplementary materials. Similarly, the number of days with freezing temperature has been decreasing over the time since data collection began in 1964 (Figure 4, *R*^2^ = 0.11, *F*_1, 47_ = 6.71, *p* < 0.013). Prior to 1984, there were no documented years where temperatures dropped below freezing, but since 2004 there are as many years without frost as there are with freezing temperature (Figure 4, Figure S4).

**Figure 5:**
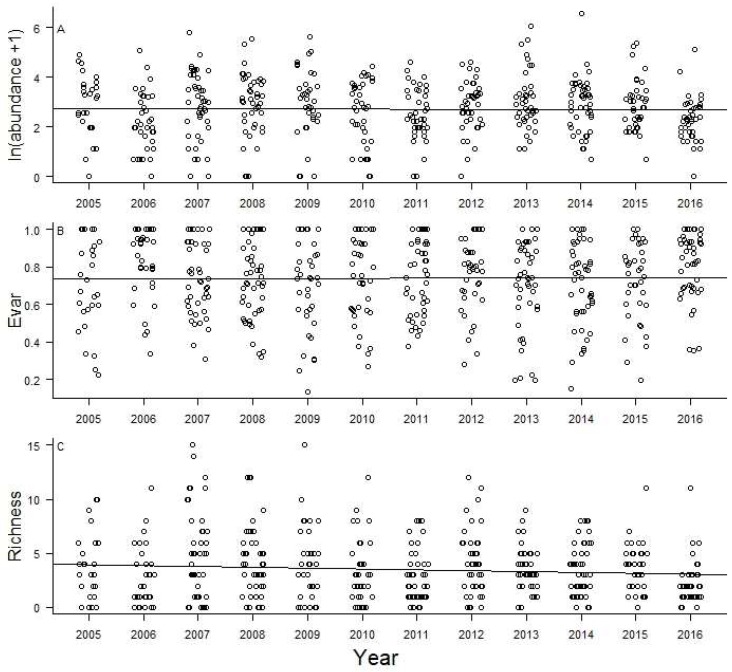
Community trends over time. Simple linear regressions showing that the overall abundance and evenness (Evar) did not change over the 12 years of sampling (**A** and **B**, respectively). However, there was a weak negative relationship between species richness and sample year (**C**). Jitter was used on *x* axis values to make overlapping points more visible.

**Figure 6:**
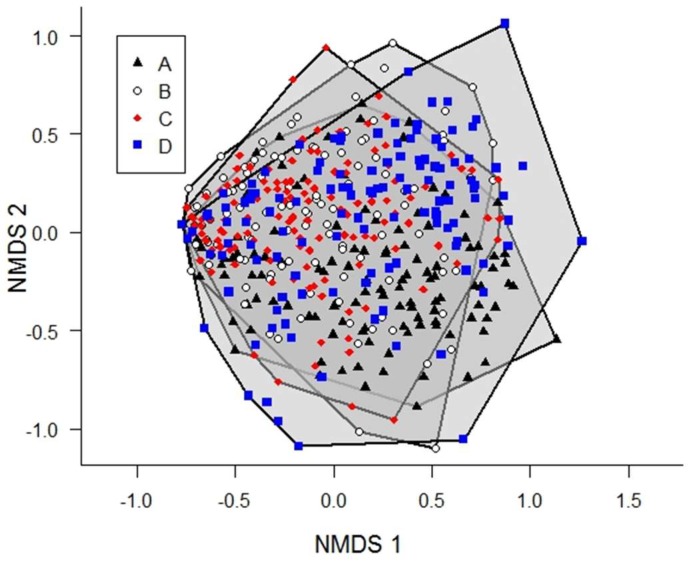
Butterfly community composition among transects. Two-dimensional non-metric multidimensional scaling (NMDS) plots suggest a very high degree of overlap among sampling routes in this study (A through D).

Butterfly abundance and evenness did not vary as a function of year during our sampling (Figure 5A,B, *R*^2^ = 0.002, *F*_1, 480_ = 0.0002, *p* = 0.99 and *R*^2^ = 0.002, *F*_1, 480_ = 0.01, *p* = 0.91, respectively). We did, however, find a weak but significant decrease in species richness over time (Figure 5C, *R*^2^ = 0.007, *F*_1, 480_ = 4.21, *p* = 0.041). NMDS indicated a high degree of overlap in butterfly community composition among sample routes (Figure 6), although no route fell completely within the others, indicating that each route had at least one unique community at some point during data collection. The overall stress for the two dimensional model visualized in Figure S5 was 31.47, which is higher than is desirable [39]; however, the results of a four-dimensional model did not differ qualitatively, and yielded an acceptable stress value of 18.01.

**Figure 7:**
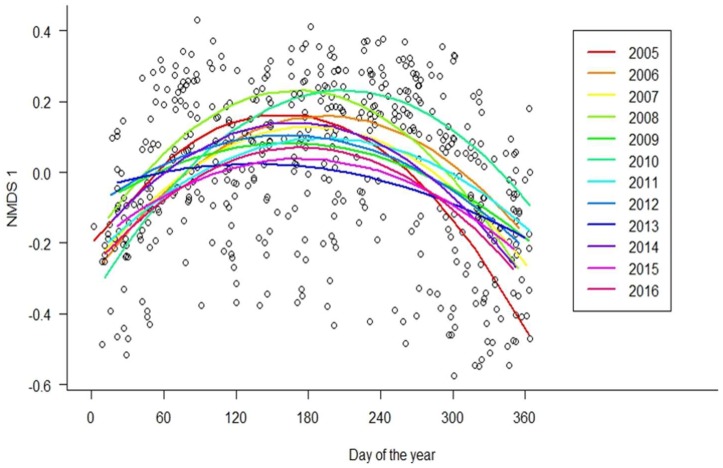
Butterfly community composition (summarized by the first non-metric multidimensional scaling axis) varied over the course of the year, as well as among years. As winter temperatures become more suitable for butterfly activity, the flight season of individual species are spreading out to take advantage of the colder months. As a result, butterfly communities are becoming less seasonal, and broad patterns of community composition are responding less strongly to the day of year as a predictor, as seen here by the flattening of the quadratic regression lines in warmer years, and roughly as the years of sampling progress. Each color represents a different sampling year, and the order follows the colors of the rainbow (i.e., 2005 is red and 2016 is purple). Notice that 2010 was a particularly cold year, and the regression line has a pronounced arch, similar to curves from earlier in the data collection, such as 2005. Conversely, 2012 and 2013 were warmer years, and have relatively little change in community composition over the course of the year. Individual points represent all sampling bouts, unseparated by year.

**Figure 8:**
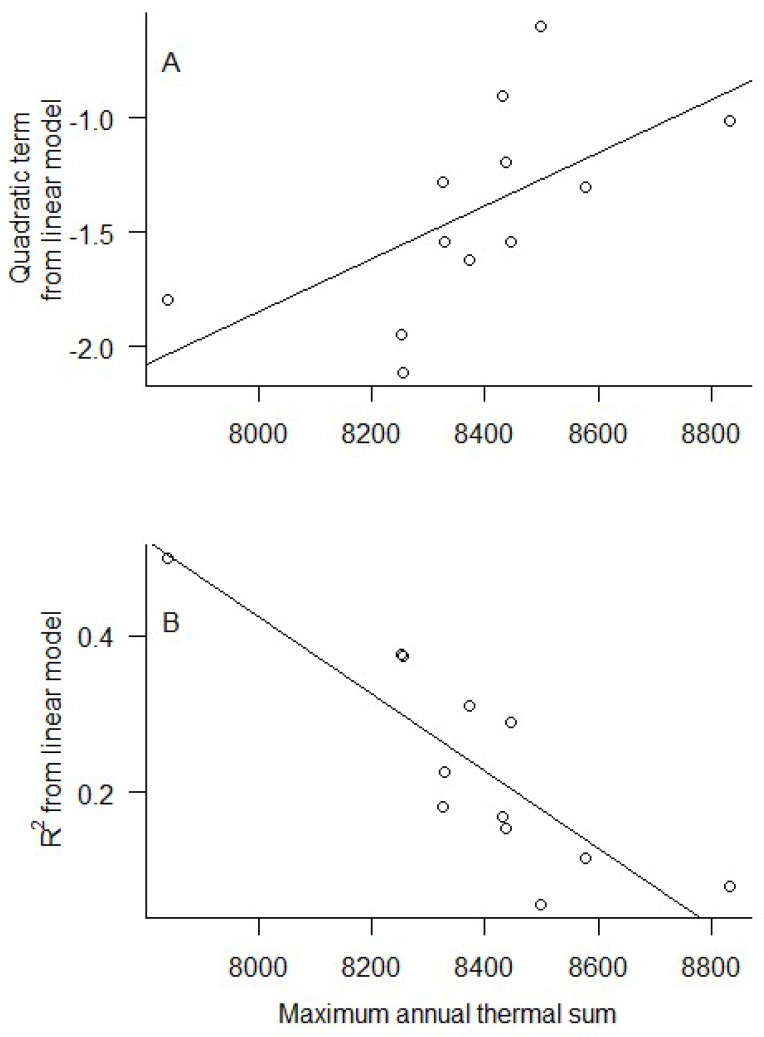
Thermal sum moderates the fit and predictability of seasonality models. Simple linear regressions showing how terms from the linear model vary as a function of thermal sum. In warmer years (i.e., years with greater thermal sum values), butterfly communities are more homogenous and less seasonal; thus, the community composition data vary less as a function of day of the year, here by the quadratic term (**A**) and *R*^2^ (**B**) from annual polynomial regressions of community composition (NMDS) as a function of day of the year.

The best-fit model for explaining community composition included the day of the year and day of the year^2^ as fixed effects, and route as a random effect (Table 1 and Table 2). The next-best model was identical but included lag precipitation, although precipitation appears to be a pretending variable, and this model was therefore discarded (Table 1). When analyzed separately for each year (Figure 7), the day of the year was a strong predictor of community composition (NMDS 1) for every year tested except two. Furthermore, the quadratic terms of the regression models and the R^2^ varied as a function of annual thermal sum (Figure 8, *R*^2^ = 0.31, *F*_1, 10_ = 5.95, *p* = 0.035 and *R*^2^ = 0.69, *F*_1, 10_ = 25.58, *p* < 0.0005, respectively). Prior to performing the mixed models, we calculated Moran’s I as a measure of spatial autocorrelation and determined that the data are spatially autocorrelated (observed = −0.079, expected = −0.002, *p* < 0.0001).

## 4. Discussion

The twelve years of data included in the present analyses succeeded in capturing natural annual variation in the abiotic environment and its impact on a butterfly community. We found that temperature (thermal sum) impacted the phenologies of butterflies in our study system, but that the phenological sensitivities to temperature varied among species. This gradient of thermal sensitivity caused a lull in butterfly activity mid-winter in cold years, resulting in a later flight season for some early species and greater overlap with later-season species. For example, the common buckeye (*Junonia coenia*), which overwinters as an adult and is strongly polyphenic, was relatively common throughout the year regardless of temperature, and observations of the gulf fritillary (*Agraulis vanillae*), a migratory species, were similarly less sensitive to colder temperatures. Likewise, the phaon crescent (*Phyciodes phaon*) tends to be an opportunistic breeder as long as favorable conditions allow. On the other hand, Henry’s elfin (*Callophrys henrici*), which overwinters as a pupa and has a single spring flight, was slower to emerge in colder years. Similarly, the zebra longwing (*Heliconius charithonia*), a subtropical species and more sensitive to frost, was reported later in our surveys in colder years.

The observed changes in community composition were driven entirely by relative changes in phenologies, suggesting that climate change will have immediate impacts on butterfly communities through changes in temporal co-occurrence patterns. Specifically, community composition differed the most between mid-summer and either early or late season in the colder years, and the warmer years were dramatically less seasonal. In addition to patterns driven by developmental sensitivities to temperature, frost sensitivity could also play a role. Although a trend of fewer frost events over time was not captured in our study period, longer-term data from our area suggests that hard frosts are becoming less frequent, a trend that is expected to continue due to climate change [40,41]. Our study therefore suggests that butterfly community composition will continue to change over time as the climate warms, even in the absence of overall changes in species richness and evenness.

Our results support observations that butterfly emergence is advancing over time, likely due to climate change [42]. Our results also support findings that winter and spring temperatures affect species phenologies in unique ways [15,43], which may have driven the observed changes in temporal overlap in the present study. Our study is notable, however, in that standard descriptors of community composition, such as abundance, evenness, and species richness, did not vary over time. Our study is also unique in showing how regions without strongly demarcated seasons (e.g., sub-tropical and tropical areas) may be more sensitive than temperate areas, because species can drift forward or backward. For example, warm winters could cause late-season species to persist longer into the year, and early-season species to emerge earlier, to the point that they could overlap in time. One caveat that should be acknowledged, however, is that the four sites are close in proximity to one another, and in some cases are within the foraging range of many of the sampled species. Thus, our design suffers from spatial autocorrelation. We have controlled for spatial autocorrection with appropriate mixed models, but our inference space could be limited as a result. Nonetheless, the observed patterns are logical, and we are confident in our interpretation of the data.

Despite the numerous studies documenting emerging phenological mismatches as a response to climate change [44], few studies focus on communities of ecologically similar taxa within trophic levels. However, most ecological systems are composed of such communities, and species rarely interact in an isolated pair-wise fashion. Thus, the effects of factors affecting whole communities should not be overlooked. The types of changes in community composition demonstrated in this study could have fitness consequences on both the insects and the plants that they visit. Butterflies and other flower-visiting insects play a critical role in the ecosystem function as pollinators, and changes in pollinator community composition can affect plant–pollinator interactions, competition for floral resources, pollinator visitation patterns, and plant reproduction.

## 5. Conclusions

Our results suggest that climate change is already affecting ecological communities, and in ways that may go undetected when only looking at abundance, richness, and evenness. We found that butterfly communities were responding in more subtle ways, with the slight drifting of species specific phenologies compounding into different communities being present through the season. Such changes could have fitness consequences by altering competition for resources, as well as indirect effects mediated by species interactions across trophic levels. We therefore recommend that community ecology focused on climate change use long-term research whenever possible, and that experimental designs be created that capture as much within-season variation in addition to interannual variation.

## Figures and Tables

**Table 1:** AICc table for fixed effects.

Model	*K*	AICc	ΔAICc	*w_i_*	c*w_i_*	Log *L*
doy + doy^2^	4	−162.46	0	0.73	0.73	85.27
doy + precipitation + doy^2^	5	−160.43	2.03	0.27	1	85.28
Precipitation × doy^2^	4	−95.4	67.06	0	1	51.74
Precipitation	3	−75.01	87.45	0	1	40.53
doy	4	−73.88	88.58	0	1	40.98
doy^2^	3	−70.71	91.76	0	1	38.38
precipitation + precipitation^2^	3	−56.8	105.66	0	1	31.43

Table comparing the fixed effects of *a priori* models of butterfly community composition (NMDS 1). *K* = number of parameters in the model, *w_i_* = Akaike weights, c*w_i_* = cumulative Akaike weights, and log *L* = log likelihood. Model parameters: “doy” is the day of year, and “precipitation” is the total precipitation in the 60 days prior to the sample bout. Fixed effect model structure was determined first, and random effect model structure was determined afterward (Table 2). Parameter estimates for the best fit model are found in the Results.

**Table 2:** AICc table for models with fixed and random effects.

Model	*K*	AICc	ΔAICc	*w_i_*	c*w_i_*	log *L*
route	5	−378.46	0	0.54	0.54	194.29
effort + route	6	−376.42	2.04	0.19	0.74	194.3
route + temperature	6	−376.41	2.05	0.19	0.93	194.29
effort + route + temperature	7	−374.36	4.1	0.07	1	194.3
effort	5	−148.75	229.71	0	1	79.44
effort + temperature	6	−146.7	231.77	0	1	79.44
temperature	5	−145.55	232.91	0	1	77.84

Table comparing the random effects of *a priori* models of butterfly community composition (NMDS 1) using a constant set of fixed effects, previously chosen to be the best-fit model (Table 1). *K* = number of parameters in the model, *w_i_* = Akaike weights, c*w_i_* = cumulative Akaike weights, and log *L* = log likelihood. Model parameters: “route” is the sample route (A through D), “effort” = duration of the sampling bout in minutes, and “temperature” is the temperature at the beginning of the sample bout in °C. The fixed-effect model structure was determined first, and the random-effect model structure was determined afterward. Parameter estimates for the best-fit model are found in the Results.

## Supplementary Materials

The following are available online at http://www.mdpi.com/2075-4450/9/4/174/s1, Figure S1: Species accumulation curve; Figure S2: Rarefication curve; Figure S3: Thermal sum over time; Figure S4: Frost events described by Disney Horticulture; Figure S5: Stress values; Table S1: Butterfly species list and relative abundances; File S1: Data used for analyses.
