# Supplementary material for: Drifting Phenologies Cause Reduced Seasonality of Butterflies in Response to Increasing Temperatures
Source: Insects. 2018 Nov 30;9(4):174. doi: 10.3390/insects9040174 (PMC6317056; doi:10.3390/insects9040174)
Supplement: Supplementary file 1 [file insects-09-00174-s001.zip › Supply/Figures.docx]

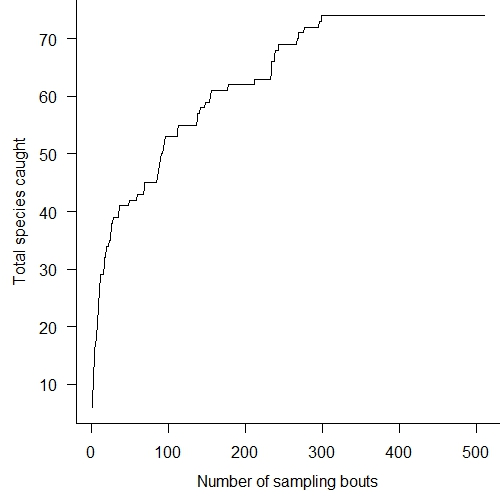


**Figure S1.** Species accumulation curve. A species accumulation curve reveals a plateau after 299 sampling bouts, indicating that our sampling effort adequately characterized the species richness of our study area.


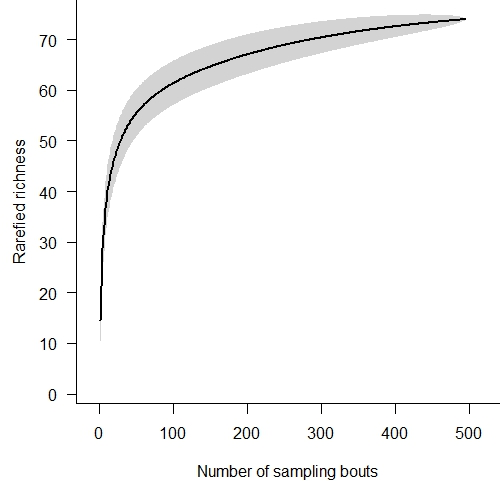


**Figure S2.** Rarefication curve. A rarefied richness + 1SE as a function of sampling effort reveals a saturating function, indicating that our sampling effort adequately characterized the species richness of our study area.


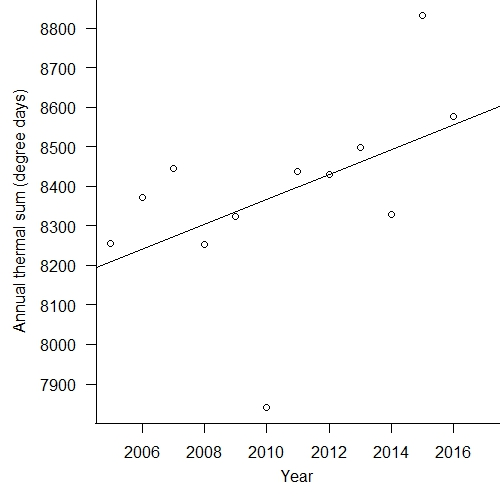


**Figure S3.** Thermal sum over time. The thermal sum increased over the course of our sample period, although the polar vortex in 2010 is an influential outlier, and the relationship is not significant (Figure 3).


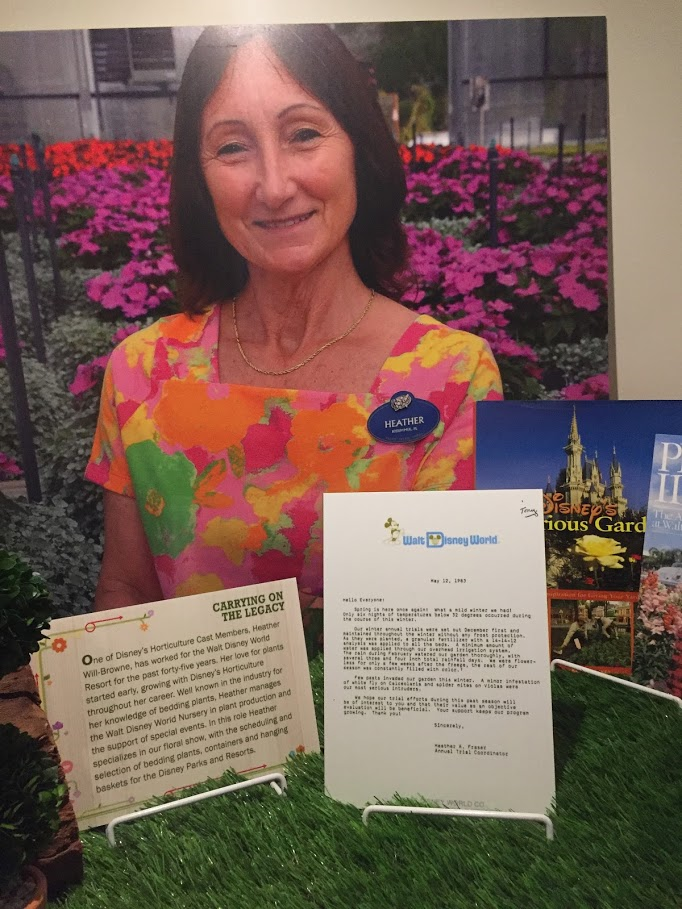


**Figure S4.** Frost events described by Disney Horticulture. Letter from long time Walt Disney World Horticulture team member Heather Will-Browne describing a flowering plant trial in the winter of 1982, which she described as “mild” after only 6 nights of freezing temperatures. The full text of her letter reads “May 12, 1983—Hello Everyone: Spring is here once again! What a mild winter we had! Only six nights of temperatures below 32 degrees occurred during the course of this winter. Our annual winter trials were set out December first and maintained throughout the winter without any frost protection. As they were planted, a granular fertilizer with a 14-14-12 analysis was applied to all the beds. A minimum amount of water was applied through our overhead irrigation system. The rain during February watered our garden thoroughly, with several three and our inch total rainfall days. We were flowerless for only a few weeks after the freeze, the rest of tour season was consistently filled with color. Few pests invaded our garden this winter. A minor infestation of white fly on Calceolaria and spider mites on Violas were our most serious intruders. We hope our trial efforts during this past season will be of interest to you and that their value as an objective evaluation will be beneficial. Your support keeps our program growing. Thank you! Sincerely, Heather A. Fraser, Annual Trial Coordinator”.


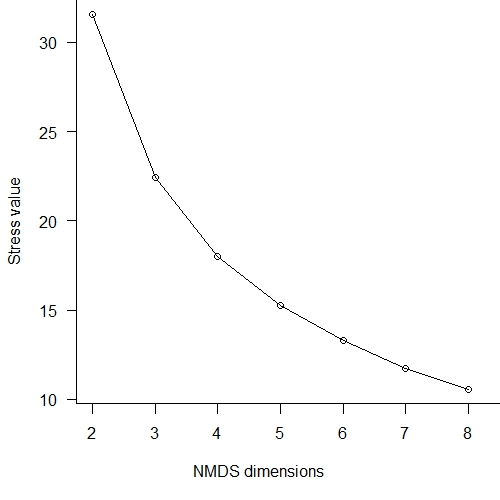


**Figure S5.** Stress values. The final non-metric multidimensional scaling (NMDS) model included four dimensions, and yielded a stress value of 18.01.
